# Estrogen Signaling and the DNA Damage Response in Hormone Dependent Breast Cancers

**DOI:** 10.3389/fonc.2014.00106

**Published:** 2014-05-14

**Authors:** C. Elizabeth Caldon

**Affiliations:** ^1^Genome and Replication Stability Group, The Kinghorn Cancer Centre, Garvan Institute of Medical Research, Sydney, NSW, Australia; ^2^St Vincent’s Clinical School, Faculty of Medicine, UNSW Australia, Sydney, NSW, Australia

**Keywords:** estrogen receptor, DNA damage response, breast cancer, p53, BRCA1, DNA repair, tamoxifen, DDR

## Abstract

Estrogen is necessary for the normal growth and development of breast tissue, but high levels of estrogen are a major risk factor for breast cancer. One mechanism by which estrogen could contribute to breast cancer is via the induction of DNA damage. This perspective discusses the mechanisms by which estrogen alters the DNA damage response (DDR) and DNA repair through the regulation of key effector proteins including ATM, ATR, CHK1, BRCA1, and p53 and the feedback on estrogen receptor signaling from these proteins. We put forward the hypothesis that estrogen receptor signaling converges to suppress effective DNA repair and apoptosis in favor of proliferation. This is important in hormone-dependent breast cancer as it will affect processing of estrogen-induced DNA damage, as well as other genotoxic insults. DDR and DNA repair proteins are frequently mutated or altered in estrogen responsive breast cancer, which will further change the processing of DNA damage. Finally, the action of estrogen signaling on DNA damage is also relevant to the therapeutic setting as the suppression of a DDR by estrogen has the potential to alter the response of cancers to anti-hormone treatment or chemotherapy that induces DNA damage.

## DNA Damage Induced by Estrogen

Lifetime exposure to estrogen is a major risk factor for breast cancer. Elevated serum levels of estrogen are associated with a 2–2.5× greater risk of breast cancer development (1) and high levels of estrogen in the breast of postmenopausal women are associated with increased cancer risk (2). Estrogen signaling drives proliferation in the 60–70% of breast cancers that express the estrogen receptor, and adjuvant anti-estrogen therapy is prescribed to the majority of these patients to prevent breast cancer recurrence.

Estrogen signals through its two receptors, estrogen receptor α (ERα) and estrogen receptor β (ERβ). Only ERα is essential for breast development and activates pro-proliferative signaling in the normal breast and breast cancer, whereas ERβ generally antagonizes ERα in the breast (3). Upon estrogen binding ERα acts by parallel pathways to alter gene expression. ERα translocates to the nucleus to activate gene targets directly or in cooperation with co-activator proteins, or it can transactivate growth receptors to boost receptor tyrosine kinase signaling. These pathways converge to promote growth and proliferation and suppress apoptosis (3).

Despite the risks associated with estrogen exposure the exact mechanisms by which estrogen contributes to the initiation and progression of breast cancer remains elusive. However, a major mechanism is potentially the induction of DNA damage as estrogen treatment leads to double stranded DNA breaks and genomic instability (1, 4, 5). Early breast cancer lesions exhibit chromosomal instability and aneuploidy (6), and in rat models this is linked to estrogen exposure (7). Estrogen can induce DNA damage via the production of oxidative metabolites that cause DNA adducts, or other oxidative DNA damage, and this is supported by *in vitro* and animal model studies (1). The second explanation for estrogen-induced DNA damage is that hyperactivated estrogen signaling provokes excessive proliferation when pathways become dysregulated, and this theory has strong support from *in vitro* modeling and gene signatures in breast cancer (3). Excessive proliferation promotes DNA damage accumulation due to insufficient timely repair leading to replication fork stalling and possibly even double stranded DNA breaks (8). It is likely that both carcinogenic estrogen metabolites and deregulated estrogen signaling contribute to estrogen-induced DNA damage. In this perspective a third possibility is raised, that estrogen signaling suppresses the DNA damage response and DNA repair to allow the accumulation of genomic change conducive to tumorigenesis.

## DNA Damage Response and DNA Repair Pathways Altered by Estrogen Signaling

DNA damage is recognized and processed by series of pathways called the “DNA damage response (DDR)”. The DDR assesses the scope and severity of DNA damage to initiate cell cycle arrest, senescence, repair, or in the case of irreparable damage, apoptosis. If repair is activated then a number of different repair mechanisms can be engaged [reviewed in Ref. (9)]. Small lesions of damaged or incorrectly inserted nucleotides are repaired by base excision repair (BER), nucleotide excision repair (NER), or mismatch repair (MMR). The more catastrophic double stranded breaks are repaired via non-homologous end-joining (NHEJ) or homologous recombination (HR). Small distorting lesions are extremely common so the pathways that repair these defects (BER, NER, and MMR) are also activated by constant genome surveillance, and repair is coupled to transcription and DNA replication.

The DDR signals through three main effector kinases, ATM, ATR, and DNA-PK. ATM and DNA-PK recognize double stranded breaks whereas ATR responds to single stranded regions that occur at stalled replication forks and double stranded break overhangs. The signaling pathways downstream of ATM, ATR, and DNA-PK involve a myriad of proteins, however there are a number of key effector proteins that include CHK1, CHK2, BRCA1, 53BP1, and MDC1 which signal to DNA repair coordinators such as BRCA2, PALB2 and to cell cycle checkpoints and the apoptotic machinery. The major tumor suppressor protein, p53, is activated downstream of ATM/ATR, and acts as a genome guardian to determine whether cells should arrest or apoptose. There is significant cross-talk between the various pathways depending on the nature and severity of the DNA damage.

**Figure 1:**
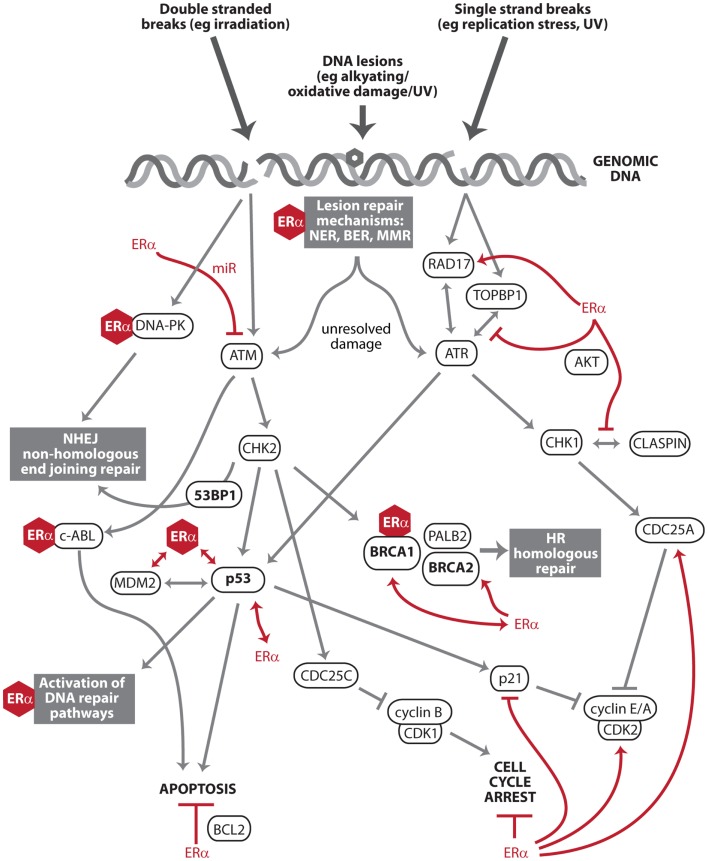
**Key effectors of the DNA damage response and DNA repair that intersect with estrogen receptor α signaling**. The DNA damage response (DDR) is a series of pathways that recognize and process DNA damage. After DNA damage recognition, signals are transduced and amplified through kinase activation (ATM, ATR, DNA-PK, CHK1, and CHK2) to downstream effectors (e.g., p53 and BRCA1) that facilitate DNA repair, apoptosis, and cell cycle arrest. Estrogen receptor α (ERα) exists in complex with multiple members of the DDR and DNA repair pathways (e.g., DNA-PK, BRCA1, p53, and MDM2). These protein:protein interactions are denoted by ERα represented as a hexagon. This includes c-Abl, a multi-functional regulator of the DDR and its downstream pathways (14). ERα also transcriptionally regulates or is regulated by other members of these pathways (e.g., ATM, ATR, CHK1, BRCA2, and DNA damage checkpoint protein Rad17), denoted by red lines. ERα signaling antagonizes two major endpoints of DDR action: apoptosis and cell cycle arrest (red lines).

The DDR is important to estrogen carcinogenesis as it dictates how estrogen-mediated damage is processed by breast cells. In prior genome wide studies of estrogen action, the major regulatory nodes of the ERα transcriptional program have included proliferation, growth, and apoptosis, but not the DDR or DNA repair (3). However, there is a growing body of literature, which identifies estrogen signaling as regulating key effector DDR proteins such as ATM, ATR, p53, BRCA1, and BRCA2, as well as direct interactions with the DNA repair machinery. This is significant not only for estrogen carcinogenesis, but also for the processing of any genotoxic insults by estrogen-responsive tissues. Described below are the most important interactions between ERα, the DDR, and DNA repair pathways (Figure 1). ERβ is not discussed in this perspective, but it should be noted that ERβ has opposing effects to ERα in many contexts (10), and this is also true of regulation of the DDR and DNA repair (11–13).

### Regulation of effector kinases ATM, ATR, and DNA-PK

ATM and ATR are key initiators of the DDR, and both are negatively regulated by ERα. ERα downregulates transcription of ATM via the activation of *miR-18a* and *miR-106a* (11). The ATR/CHK1 signal transduction cascade is suppressed by ERα-transactivated AKT phosphorylation of TOPBP1 to prevent an interaction with ATR at sites of DNA damage (15). AKT also phosphorylates CHK1 to prevent its interaction with co-activator CLASPIN (15). The downregulation of ATM and ATR by ERα interferes with the induction of cell cycle checkpoints so that cells continue to progress through the cell cycle after DNA damage, and DNA repair is delayed or not engaged (15, 16). Estrogen activity does not, however, preclude activation of the DDR. γ-H2Aχ foci form in response to estrogen-induced DNA damage, and the co-localization of Rad51 to these foci suggests the activation of HR (4).

While ERα negatively regulates both ATM and ATR, it is possible that ERα positively regulates DNA-PK mediated repair based on recent findings of DNA-PK regulation by the androgen receptor (AR). AR regulation of DNA-PK catalytic subunit (DNA-PKcs) promotes the repair of DNA double stranded breaks and resistance to DNA damage and DNA-PKcs likewise potentiates the function of AR (17). Like AR, ERα is in a complex with DNA-PK (18) and ERα is stabilized and its transcriptional function potentiated by DNA-PK (19), and by analogy to AR, ERα may also transactivate DNA-PK.

If ERα does positively regulate DNA-PK, ERα may suppress DNA repair processes of higher fidelity (ATM- and ATR-mediated) in preference for DNA-PK-mediated NHEJ. This is consistent with observations of ERα activity leading to the accumulation of DNA damage (1) as it would sustain proliferation by not engaging the ATM/ATR pathways, while promoting DNA-PK-mediated NHEJ to maintain genome integrity. Toillon et al. found that estrogen treatment of irradiated breast cancer cells led to their sustained proliferation without any increase in p53 activation or apoptosis (20). This is consistent with a failure to activate ATM or ATR but the repair of DNA by DNA-PK mediated NHEJ.

### BRCA1

BRCA1 is a downstream effector of the DDR that is recruited to sites of DNA damage, functions directly in HR, but also influences cell cycle arrest and other DNA repair pathways. There is strong evidence that BRCA1 limits estrogen-mediated tumorigenesis: *Brca1* knockout mice show an enhanced proliferative response to estrogen treatment and accelerated development of preneoplastic mammary lesions (21), and the reduction of serum estrogen levels by oophorectomy protects carriers of the *BRCA1* mutation against breast cancer (22). Indeed, BRCA1 has a negative effect on ERα, through direct binding to inhibit ERα-mediated gene transcription (23, 24), downregulation of ERα co-activator, p300 (25), reduced cross-talk from growth factor signaling (26), and potentially monoubiquitination (25, 27). These effects are antagonized by cyclin D1, a direct transcriptional target of ERα that is instrumental in estrogen-induced proliferation (28).

While BRCA1 suppresses ERα, ERα regulation of BRCA1 enhances BRCA1 function. Estrogen promotes transcription of *BRCA1* via binding of an ERα/p300 complex (29), and stimulates the formation of a complex between ERα, CBP, and BRCA1 that facilitates double stranded break repair (30). Surprisingly, BRCA1 induces the transcription of *ESR1* which encodes ERα, and the positive feedback between BRCA1 and ERα provides a rational explanation for why many BRCA1 negative cancers are ERα negative (31).

### p53

Estrogen receptor α and p53 have a bi-directional relationship affecting both expression and function. The *TP53* gene is transcriptionally activated by ERα (32, 33) and downstream of ERα-target, c-MYC (34), and ERα stabilizes the p53 protein (35). Despite ERα inducing higher levels of p53 it may not be active: in breast cancer cell lines estrogen induces cytoplasmic redistribution of p53 to reduce its transcriptional function (12, 36). ERα alters the p53 transcriptional program to reverse transcriptional activation and repression by p53, including downregulation of the p53-mediated apoptotic response induced by DNA damage (37). ERα represses p53-mediated transcription either through the recruitment of co-repressors (38) or via independent targeting and repression of p53 target gene sets (39). A separate subset of target genes for p53 activation is enhanced by ERα activity (37).

p53 and ERα exist in complex with MDM2, and this complex modulates the activity of p53 and ERα. MDM2 is a negative feedback regulator of p53 (40), whereas MDM2 positively regulates ERα transcriptional activity, most probably through direct MDM2:ERα interaction (41, 42). Conversely, the MDM2/p53/ERα ternary complex downregulates the activity of ERα by monoubiquitination, probably via the ubiquitin ligase activity of MDM2 (43). MDM2 may also downregulate ERα independently of p53 (43). In the presence of cellular stress, including UV-mediated DNA damage, p53 dissociates from MDM2 and this is associated with an increase in ERα levels and block of the estrogen-dependent downregulation of ERα (43). Paradoxically, while ERα represses p53-mediated transcription, ERα also protects p53 from repression by MDM2 (40), and estrogen treatment is necessary for a p53 response to be mounted in the mouse mammary gland against ionizing radiation (44).

p53 upregulates the expression of *ESR1*, but alters the transcriptional functions of ERα. p53 induction of *ESR1* occurs following DNA damage such as irradiation (45). Like ERα modulation of p53 function, p53 alters the transcriptional program of ERα to repress certain estrogen responsive genes such as *BRCA2*, *c-JUN*, and *BCL2* (37, 46). Indeed it appears that the combination of ERα and p53 induces a distinct transcriptional program compared to either ERα or p53 alone (47).

Overall this body of work suggests that estrogen and ERα upregulate but sequester p53, such that the DDR and DNA repair are suppressed in the presence of active estrogen signaling, but there is still some safeguard via p53. When estrogen treated breast cancer cells are irradiated there is partial activation of p53 and its downstream pathways, but the pro-proliferative effects of estrogen override any checkpoint-mediated cell cycle arrest (20). Conversely, in mouse models, p53 provides protection from lymph node hyperplasia and ductal carcinoma *in situ* (DCIS) induced by deregulated estrogen signaling (48).

### DNA repair machinery

Estrogen receptor α interacts directly with DNA repair proteins with varying impact on DNA repair mechanisms and ERα function. This includes FEN1, MPG, APE1, and TDG of the BER pathway (49, 50), *O*(6)-methylguanine-DNA methyltransferase, which corrects mutagenic DNA lesion *O*(6)-methylguanine back to guanine (51), NHEJ repair proteins Ku70 and Ku86 in the context of gene transcription (18) and MSH2 of the MMR pathway (52). The binding of ERα to MPG enhances BER (53), while estrogen treatment upregulates or downregulates NER, depending on cell type (54, 55). The binding of repair proteins has different outcomes on ERα: MPG inhibits ERα-induced transcription and transactivation of signaling pathways (53), MSH2 and TDG transactivate ERα (50, 52), and the binding of FEN1 and APE1 to ERα has distinct effects on different ERα target genes (56, 57).

Estrogen receptor α interacts with other core DNA damage processing proteins, although the consequence for DNA repair or ERα action is unknown. Estrogen treatment upregulates *BRCA2* (58) of the HR pathway, and through phosphorylation protects BRCA2 from degradation (59). ERα also directly interacts with DNA repair signaling and processing protein PARP-1 in the context of ERα-mediated gene transcription (18), which potentially affects ERα-regulated gene networks.

### Cell cycle checkpoints and apoptosis

One of the most important functions of the DDR is to halt proliferation via the activation of cell cycle checkpoints or induce apoptosis. The effector proteins of these responses are not only targets of the DDR but as a set are antagonized by pro-proliferative ERα signaling. The DDR induces a G_1_/S phase arrest downstream of ATR via CDC25A inhibition of cyclin A/E/CDK2 complexes, and downstream of p53 via p21 inhibition of cyclin D/CDK4/6 and cyclin E/CDK2 complexes. A G_2_/M arrest is induced downstream of Chk1/Chk2 via activation of CDC25 phosphatases to inhibit cyclin B/Cdk1 complexes (60). ERα antagonizes cell cycle arrest by upregulating *CCND1* (cyclin D1), *CCNE2* (cyclin E2), and *CDC25A*, and downregulating *CIP1* (p21) downstream of c-MYC (61–63). Likewise, p53 induces apoptosis by induction of *FAS-R*, *BAX*, *PUMA*, and *NOXA* (64), but ERα induces an anti-apoptotic signal including upregulation of *BCL2* (65).

Consequently, active ERα signaling will antagonize the anti-proliferative and pro-apoptotic signals of the DRR. The outcome will be dictated by the strength of each signal, but ERα signaling is able to sustain proliferation in situations where otherwise DNA damage would have induced a cell cycle arrest and apoptosis (15, 20, 66).

## Disruption of DDR and DNA Repair Pathways in Breast Cancer, and Their Association with ERα Status and Prognosis

DNA damage pathways are altered in breast cancer by mutation, changes in expression, amplification, and methylation, and as a class the DDR and DNA repair proteins are frequently altered in cancer and associated with poor prognosis. A survey of the literature shows that DDR pathways differ significantly between ERα positive and ERα negative breast cancer (Table 1). At least part of this change may be due to loss of ERα signaling, and certainly changes to p53, ATM, and *TIMELESS* (which functions in the ATR pathway) are consistent with the loss of ERα regulation of these genes/proteins. However, given that changes to DNA damage processing are a hallmark of cancer that contributes to tumor initiation, some of the changes no doubt precede loss of ERα, and may in fact contribute to its loss. This is exemplified in cancers with low BRCA1 and ERα, and BRCA1 loss is hypothesized to lead to ERα downregulation in breast cancer (31). Nevertheless, the presence or absence of DDR/DNA repair proteins will affect DNA repair in hormone-responsive cancers and the bi-directional regulation of the DDR/DNA repair and ERα. Likewise, the loss of ERα will affect the DDR/DNA repair in ERα negative cancers.

**Table 1:** **DNA damage response and DNA repair genes altered in breast cancer and relationship to ERα status**.

Gene/protein	Interaction with ERα	Alteration and relationship to ERα status in breast cancer	Prognosis	Reference
ATM	ERα downregulates *miR-18a* and *miR-106a* to downregulate ATM protein expression, and *miR-18a* directly binds to the ATM-3′-UTR	ATM protein is higher in ER negative breast cancers	High ATM protein is correlated with recurrence in breast cancer	(11, 16, 67)
ATR	ATR is functionally downregulated by ERα transactivated AKT signaling, which suppresses the DNA damage induced association between ATR:TOPBP1	–	–	(15)
BRCA1	The BRCA1:Oct1 complex directly binds the *ESR1* promoter to drive ERα transcription; BRCA1 suppresses ERα-mediated transcription through direct binding and co-activators; ERα promotes *BRCA1* transcription via an ERα/p300 transcriptional complex	Low *BRCA1*/BRCA1 (by mutation, methylation, or low mRNA) is associated with ER negative breast cancers	Oophorectomy (resulting in reduced estrogen levels) is protective against breast cancer in *BRCA1* familial breast cancers	(22–26, 29, 31)
*BRCA2*	*BRCA2* is upregulated by estrogen treatment, possibly as an indirect target rather than via ERα	*BRCA2* is higher in ER negative breast cancers	High *BRCA2* predicts poor disease-free survival	(68, 69)
c-ABL	c-ABL enhances estrogen receptor ERα transcriptional activity through its ERα stabilization by phosphorylation	Expression of c-ABL and ERα are not correlated	Co-expression of c-ABL and ERα is associated with advanced tumor stage and lymph node involvement	(70, 71)
*CHEK2*	–	*CHEK2* mutated breast cancers tend to be ERα positive	In ER positive breast cancers, *CHEK2* mutation is associated with increased risk of death and second breast cancers, but not in ER negative cancers	(72, 73)
CHK1	CHK1 is phosphorylated via ERα transactivated AKT signaling, which suppresses the DNA damage induced CLASPIN:CHK1 interaction	*CHK1* mRNA and protein are highly expressed in ER negative	*CHK1* not prognostic for outcome metastasis in breast cancer	(15, 74)
CLASPIN	CHK1 is phosphorylated via ERα transactivated AKT signaling, which suppresses the DNA damage induced CLASPIN:CHK1 interaction	*CLASPIN* mRNA and CLASPIN protein are highly expressed in ER negative breast cancers	*CLASPIN* mRNA is not prognostic for metastasis	(15, 74)
DNA-PK	The DNA-PK:ERα protein complex increases ERα phosphorylation and reduces ERα turnover. The DNA-PK:ERα complex binds to ERα responsive gene promoters, an effect that is not dependent on DNA damage	–	–	(19)
FANCD2	–	FANCD2 protein is higher in ER negative breast cancers	–	(75)
MDM2	MDM2 interacts with ERα in a ternary complex with p53. MDM2 positively regulates ERα transcriptional activity, but downregulates overall activity through ERα monoubiquitination	High MDM2 protein is correlated with ER positive breast cancers	Low MDM2 protein is correlated with high nuclear grade and lymph node involvement	(41–43, 76)
p53	ERα upregulates *TP53* and stabilizes p53, but generally suppresses p53 transcriptional function. p53 upregulates *ESR1*, but also modulates ERα induced transcription	p53 is generally wild-type and expressed in ER positive breast cancer	*TP53* mutation or p53 mutated gene signature is prognostic for poor disease-free survival	(12, 32, 33, 35–39, 45–47, 77)
PCNA	PCNA interacts directly with ERα to modulate its transcriptional function in normally proliferating cells	–	–	(78)
RAD17	*RAD17* mRNA is upregulated by estrogen in an ERα dependent manner	*RAD17* mRNA often high in breast cancer; high RAD17 protein correlated with ER negative; *RAD17* sometimes lost in ER negative, but due to loss of 5q11 locus	High *RAD17* mRNA prognostic of increased lymph node metastasis	(79–81)
*TIMELESS*	*TIMELESS* is upregulated by estrogen, probably via ERα, and downregulated by anti-estrogens	*TIMELESS* mRNA is high in ER+ patients who have relapsed for endocrine therapy	High levels of *TIMELESS* mRNA prognostic of poor relapse-free survival for ER+ breast cancers	(82)
TOPBP1	TOPBP1 is regulated downstream of ERα transactivated AKT signaling, which suppresses the DNA damage induced association between ATR:TOPBP1	TOPBP1 expression has no relationship to ERα status	Low *TOPBP1* mRNA and high TOPBP1 protein are both associated with increased breast cancer grade	(15, 83, 84)

*–, no relationship reported*.

## Perspectives

Estrogen receptor signaling is not typically thought to influence DNA repair as the literature has focused on its classic nodes of action of proliferation, growth, and apoptosis. The evidence, however, is overwhelming that ERα signaling has an impact on DNA damage processing through its regulation of ATM, ATR, DNA-PK, p53, BRCA1, BRCA2, and the DNA repair machinery. Given that estrogen can *cause* DNA damage, this raises a vital question of how estrogen receptor signaling processes the DNA damage caused by estrogen action. For example, does it dampen damage responses in favor of continuing proliferation, or does it act as a sentinel against DNA damage? Overall, estrogen receptor activity appears to downplay the response to DNA damage while simultaneously promoting proliferation. Consequently sustained ERα signaling may be permissive of the accumulation of genomic change from low level DNA damage that contributes to tumor initiation. Some of the major effectors of the DDR (e.g., p53 and BRCA1) do have negative feedback on the estrogen receptor, as does active DNA repair. Thus in the face of serious DNA damage ERα signaling is downregulated to protect the cell from continuing proliferation, and potentially allow full engagement in the DDR.

Several critical experiments will clarify whether active ERα signaling overrides the DDR. These include co-treatment with estrogen and different DNA damaging agents to determine the extent to which the DDR is activated and how ERα promoter binding is affected by DNA damage. This should incorporate the titration of doses of DNA damage to determine if there is a tipping point between sustained proliferation due to ERα action, and engagement of the DDR and DNA repair. Since ERα has cross-talk with both BRCA1 and p53, the combinatorial effects should be considered by simultaneously activating ERα signaling and treating with DNA damage in the context of BRCA1 and p53 ablation. Finally, it is a priority to investigate the effect of ERα on its binding partners DNA-PK, PCNA, and PARP-1 in the context of DNA damage.

The role of ERα in modulating DNA damage has important clinical implications. Anti-estrogen treatment is the mainstay of adjuvant therapy for breast cancer, but the most common therapy, Tamoxifen, is itself a source of DNA damage (85), and this damage has been detected in patients and is implicated in endometrial cancer (86). Tamoxifen has agonist effects through ERα in the endometrium (87) so it is interesting to speculate that Tamoxifen therapy induces DNA damage and disturbs a balance between estrogen signaling and the DDR in the endometrium to detrimental effect. Chemotherapies and radiation therapy induce DNA damage, so ERα may suppress the DDR to reduce the efficacy of these treatments. Indeed, patients with ER positive breast cancers have significantly lower response rates to chemotherapy than those with ER negative cancers (88), and *in vitro* studies suggest this is dependent on ERα action (89–91). Co-administration of anti-estrogens and radiation therapy or chemotherapy appears to enhance therapy cytotoxicity and a likely explanation is that anti-estrogen treatment prevents pro-proliferative bypass of cytotoxicity by estrogen (66, 90). Conversely, estrogen receptor action is needed for sustained p53 expression to allow the induction of apoptosis by chemotherapeutic doxorubicin (92), and good prognosis ERα positive breast cancers generally express p53. Consequently, the pro-apoptotic arm of the DDR appears compromised in some circumstances by the complete inhibition of ERα signaling. Further understanding of the cross-talk between ERα and DNA damage processing will provide crucial information to guide drug, radiation therapy, and hormone combination treatment of breast cancer patients.

## Conflict of Interest Statement

The author declares that the research was conducted in the absence of any commercial or financial relationships that could be construed as a potential conflict of interest.
